# Morphometric measurement of lumbar pedicle in different regions: a systematic review

**DOI:** 10.1186/s13018-023-03499-w

**Published:** 2023-01-11

**Authors:** Yixi Wang, Alafate Kahaer, Wenjie Shi, Hailong Guo, Paerhati Rexiti

**Affiliations:** 1 First Clinical Medical College, Xinjiang Medical University, Urumqi, China; 2 Departments of Spine Surgery, Xinjiang Uygur Autonomous Region, The First Affiliated Hospital of Xinjiang Medical University, Urumqi, 830054 China

**Keywords:** Anatomy, Pedicle, Width, Height, Different regions

## Abstract

**Objective:**

To collect the data of pedicle width and height in different areas, and to investigate the difference and variation rule of pedicle width and height.

**Methods:**

Comprehensive search of PubMed, Ovid Medline, and Web of science databases was performed. Collected data were imported into SPSS, and one-way ANOVA test and post hoc test were used to determine whether there were statistical differences in pedicle width and height between the different regions.

**Results:**

Oceania had the largest pedicle width and height, followed by Americans. West Asian had the largest pedicle width in Asia, followed by East and Southeast Asian, and Chinese and South Asian had similar pedicle width. Different from the variation pattern of pedicle width, the pedicle height of Chinese, East and Southeast Asian and West Asian in Asian range is similar, but the pedicle height of South Asian is significantly smaller than the first three, and has statistical significance.

**Conclusions:**

People in different regions have similar patterns of variation in pedicle width and height even though they belong to different ethnic groups. This phenomenon is particularly prominent and pronounced in populations in geographically close areas, which may be related to inter-ethnic integration due to population movement between adjacent areas. There is a relationship between the morphological characteristics of the human lumbar pedicle and geographical location.

**Supplementary Information:**

The online version contains supplementary material available at 10.1186/s13018-023-03499-w.

## Introduction

With the increasing aging population, lumbar degenerative diseases have become one of the most common diseases, and conventional treatments include decompression and fusion surgery with pedicle screws as well as cortical bone trajectory (CBT) screws [1–3]. However, regardless of which screw was used, the trajectory passed through the pedicle [4, 5]. In a study of the pullout resistance of screws, the pedicle provided 60% axial pullout resistance and 80% screw stiffness [6], which suggests that the pedicle plays an important role in spinal biomechanics and postoperative recovery. Most scholars agree that pedicle width and pedicle height are the most important anatomical parameters of the pedicle [7–9]. At this stage, most scholars only focus on the measurement of pedicle width and height in a specific regional population, and no transverse comparative study has been conducted on the width and height of pedicles in different regional or national populations were found. In this study, the width and height of pedicles in different regions or countries were compared transversely to analyze whether there were differences and potential rules based on the previous studies.

## Material and methods

### Inclusion and exclusion criteria

**Fig. 1:**
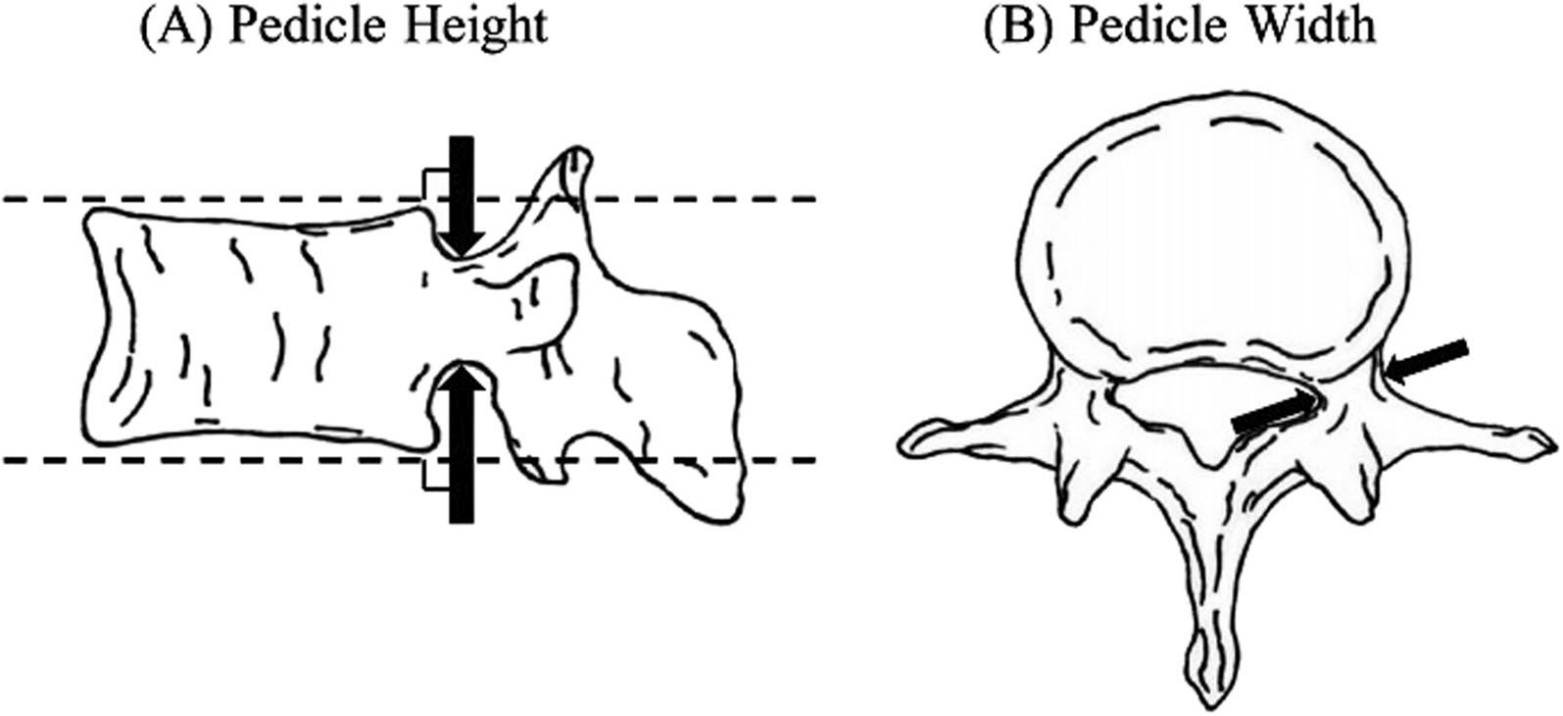
Diagram of pedicle height and width (20)

In this review, we considered the studies published as full-text articles in indexed journals, which reported the width and height of pedicle. Articles in English and Chinese with available abstract were included. No publication date limits were set. Surgical technique reports, expert opinions, letter to the editor, studies on animals, unpublished reports, review of the literature, abstracts from scientific meetings and book chapters were excluded. All retrieved articles were analyzed to determine if they provided information on pedicle height and pedicle width in detail. We defined pedicle width as the shortest distance between the medial and lateral cortex of the pedicle on the narrowest pedicle section and pedicle height as the shortest distance between the superior and inferior cortex of the pedicle on the narrowest pedicle section (Fig. 1). Articles providing pedicle width and height were further reviewed to identify the location of width and height and ensure that the data of the included articles were consistent with the definition of pedicle width and height in this study.

### Search strategy and study selection

**Fig. 2:**
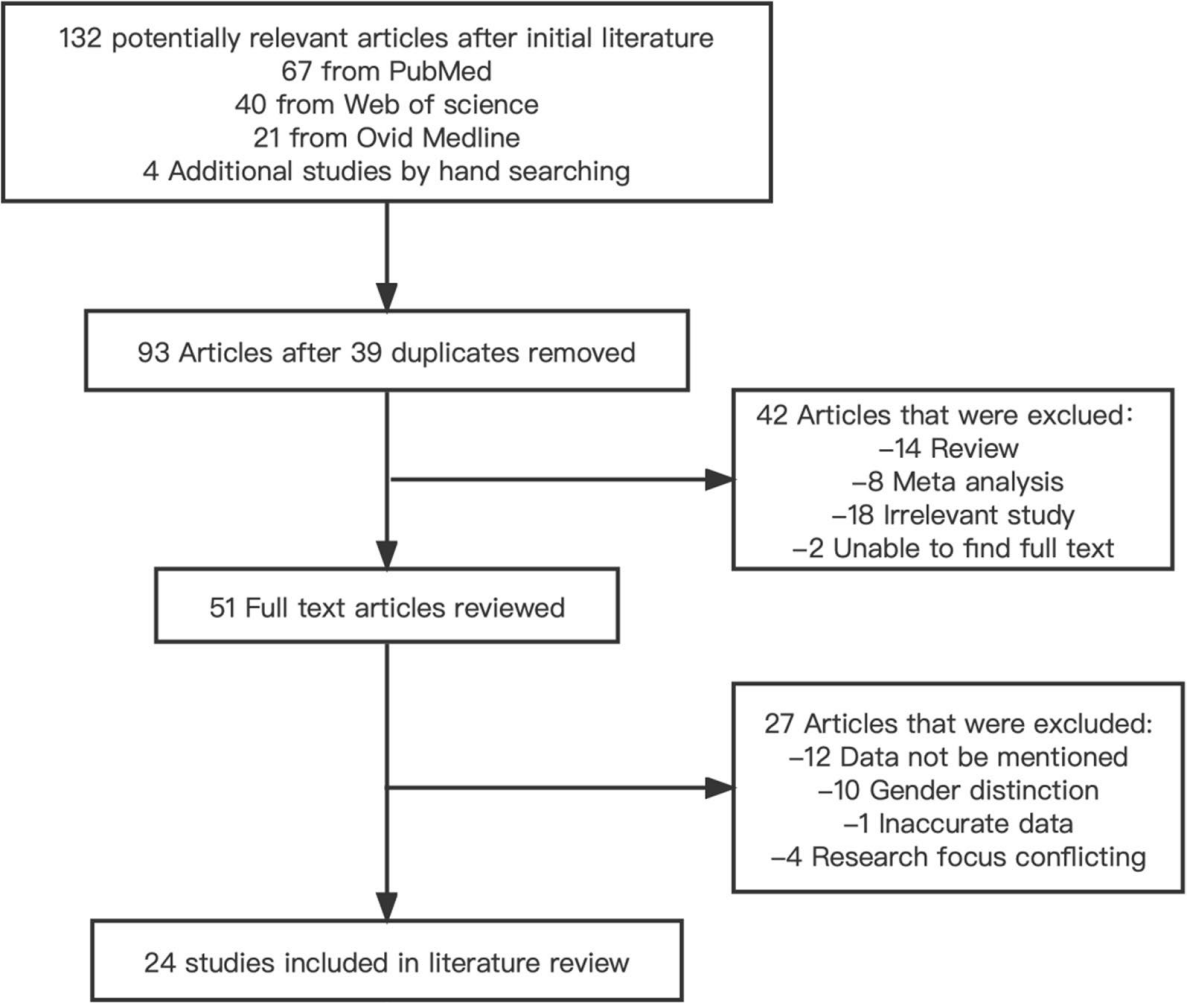
Flow diagram of literature search

A systematic review reported according to the Preferred Reporting Items for Systematic Reviews and Meta-Analyses (PRISMA) guidelines. Literature searches were conducted in the PubMed, Ovid Medline, and Web of Science databases using the following search terms: “Pedicle Lumbar”, “Pedicle Morphometry”, “Pedicle Anatomy”, and “Lumbar Vertebrae Morphometry”, as of July 2022. Flow diagram of literature search is shown in Fig. 2.

### Data extraction and analysis

The collected literatures were classified according to regions, and IBM SPSS 26 software (IBM, Armonk, NY, USA) was used for statistical analysis. First, the data were tested for normal distribution, and a one-way ANOVA test and post hoc test were used (the LSD test was used when the variances were homogeneous, and the Tamhane's T2 Test was used when the variances were uneven) to compare the width and height of L1-L5 vertebral pedicles between populations in different regions. When p < 0.05, the difference was considered significant.

## Results

24 articles were included in this study [7–30], of which 24 contained pedicle width and 18 contained pedicle height. The included articles were classified only according to their region or country and did not distinguish the age, gender, ethnicity, and measurement method of the subjects measured. 24 articles were divided into six categories according to region and country, as follows: the United States (US), China (CN), East Asia and Southeast Asia (EA and SEA), West Asia (WA), South Asia (SA), and Oceania (OA). EA and SEA include Japan and Singapore, WA includes Turkey and Israel, SA includes India and Pakistan, and OA includes New Zealand and Australia. Of the 24 articles containing pedicle, 5 were from the US [7–11], 7 from CN [12–18], 4 from EA and SEA (2 from Japan [19, 20] and 2 from Singapore [21, 22]), 3 from WA (2 from Turkey [23, 24] and 1 from Israel [25]), 3 from SA (2 from India [26, 27] and 1 from Pakistan [28]), and 2 from OA (1 from New Zealand [29] and 1 from Australia [30]), detailed data are presented in Table 1. Of the 18 articles containing pedicle height, 3 were from the US [7, 8, 10], 5 from CN [12, 14, 16–18], 4 from EA and SEA (2 from Japan [19, 20] and 2 from Singapore [21, 22]), 3 from WA (2 from Turkey [23, 24] and 1 from Israel [25]), 1 from SA (1 from Pakistan [28]), and 2 from OA ((1 from New Zealand [29] and 1 from Australia [30]), and detailed data are presented in Table 1.

**Table 1:** Summary of literature data

Study	Sample size	Vertebra	Pedicle width (mm)	Pedicle height (mm)
Zindrick et al. (US) [7]	NS	L1	8.7	15.4
		L2	8.9	15
		L3	10.3	14.9
		L4	12.9	14.8
		L5	18	14
Olsewski et al. (US) [8]	49	L1 (DM)	8.2	16.4
		L2 (DM)	8.4	15.4
		L3 (DM)	10.2	15.4
		L4 (DM)	13.2	15.4
		L5 (DM)	20.1	16.2
		L1 (RM)	8.2	18.2
		L2 (RM)	8.3	17.2
		L3 (RM)	10	16.9
		L4 (RM)	13.2	15.6
		L5 (RM)	20.1	13.8
Panjabi et al. (US) [9]	12	L1 (L)	9.2	NS
		L2 (L)	8.7	
		L3 (L)	10.1	
		L4 (L)	14.7	
		L5 (L)	19.2	
		L1 (R)	8.41	
		L2 (R)	8.46	
		L3 (R)	10.12	
		L4 (R)	12.62	
		L5 (R)	17.32	
Yu et al. (US) [10]	82	L1	8.41	15.35
		L2	8.46	14.71
		L3	10.12	14.3
		L4	12.62	13.55
		L5	17.32	13.49
	215	L1	8.48	15.68
		L2	8.63	15.02
		L3	10.37	14.55
		L4	13.14	13.68
		L5	18.25	13.49
	154	L1	8.68	16.05
		L2	8.83	15.35
		L3	10.73	14.78
		L4	13.5	14.08
		L5	18.74	13.92
	52	L1	8.45	15.84
		L2	8.67	14.96
		L3	10.6	14.62
		L4	13.37	14
		L5	18.15	14
Petrone et al. (US) [11]	141	L1	7.8	NS
		L2	7.9	
		L3	9.5	
		L4	11.2	
		L5	14.7	
	105	L1	7.2	
		L2	7.6	
		L3	9.3	
		L4	11.4	
		L5	15.2	
	86	L1	6.6	
		L2	7.2	
		L3	8.8	
		L4	10.5	
		L5	14.1	
	72	L1	6.9	
		L2	7.4	
		L3	9	
		L4	11	
		L5	14.6	
	404	L1	7.2	
		L2	7.6	
		L3	9.2	
		L4	11.1	
		L5	14.7	
Hou et al. (CN) [12]	40	L1	7	15.8
		L2	7.4	15.1
		L3	9.2	14.7
		L4	10.5	15
		L5	12.9	19.8
Chen et al. (CN) [13]	100	L1	NS	NS
		L2	8.5	
		L3	9.2	
		L4	11.9	
		L5	17	
Lien et al. (CN) [14]	21	L1 (L)	6.4	13.6
		L2 (L)	7.4	14
		L3 (L)	9.3	13.9
		L4 (L)	11.6	12.5
		L5 (L)	17.5	12.3
		L1 (R)	6.5	13.7
		L2 (R)	7	14.1
		L3 (R)	9	13.9
		L4 (R)	12.2	13
		L5 (R)	17.7	12.7
Lin et al. (CN) [15]	90	L1	5.7	NS
		L2	6.4	
		L3	7.6	
		L4	10.1	
		L5	12.6	
Guan et al. (CN) [16]	36	L1 (L)	7.8	16.3
		L2 (L)	8	15.8
		L3 (L)	9.7	15.3
		L4 (L)	10.7	16.8
		L5 (L)	12.5	22.6
		L1 (R)	7.6	16.4
		L2 (R)	7.8	15.9
		L3 (R)	9.5	15.4
		L4 (R)	10.7	16.5
		L5 (R)	12.5	22.9
Qi et al. (CN) [17]	100	L1	6.8	15.1
		L2	7.1	14.1
		L3	8.3	12.5
		L4	10.1	11.1
		L5	14.1	10.4
Ding et al. (CN) [18]	50	L1	6.4	14.1
		L2	6.6	13.3
		L3	8.3	12.8
		L4	10.1	11.5
		L5	14.5	10.6
Matsukawa et al. (Japan) [19]	100	L1	7.9	16.5
		L2	8	15.8
		L3	9.6	15.6
		L4	11.3	14.4
		L5	15.3	13.9
Morita et al. (Japan) [20]	227	L1	8.4	16.1
		L2	8.4	15.2
		L3	10.7	14.9
		L4	12.5	13.7
		L5	16.4	12.9
Tan et al. (Singapore) [21]	12	L1 (L)	6.64	15.39
		L2 (L)	7.58	15.29
		L3 (L)	8.99	14.29
		L4 (L)	10.71	15.29
		L5 (L)	13.34	20.78
		L1 (R)	6.47	15.43
		L2 (R)	7.29	14.98
		L3 (R)	8.9	15.15
		L4 (R)	10.07	15.72
		L5 (R)	13.9	20.08
Tan et al. (Singapore) [22]	10	L1 (L)	5.6	13.1
		L2 (L)	6.4	13
		L3 (L)	7.6	12.1
		L4 (L)	9.1	13
		L5 (L)	11.3	17.7
		L1 (R)	5.5	13.1
		L2 (R)	6.2	12.7
		L3 (R)	7.6	12.9
		L4 (R)	8.6	13.4
		L5 (R)	11.8	17.1
Kadioglu et al. (Turkey) [23]	16	L1 (DM)	6.4	14.2
		L2 (DM)	6.6	14.2
		L3 (DM)	8.6	13.1
		L4 (DM)	10.8	13
		L5 (DM)	12.4	13.2
	29	L1 (RM)	8.8	14.7
		L2 (RM)	9.7	14.5
		L3 (RM)	10.3	13.6
		L4 (RM)	10.8	13.6
		L5 (RM)	14.6	13.4
Torun et al. (Turkey) [24]	14	L1	8.4	14.8
		L2	8.6	15.3
		L3	10.6	14.4
		L4	12.2	13.8
		L5	17.1	14.2
Wolf et al. (Israel) [25]	55	L1	5.6	15.1
		L2	7.7	14.8
		L3	8.9	14.5
		L4	11.4	14.8
		L5	13.7	15.6
Acharya et al. (India) [26]	50	L1	7.2	NS
		L2	7.62	
		L3	8.97	
		L4	11.12	
		L5	13.91	
Mohanty et al. (India) [27]	135	L1	7.2	NS
		L2	7.6	
		L3	8.4	
		L4	10.1	
		L5	13	
Alam et al. (Pakistan) [28]	49	L1	6.1	10.2
		L2	6.6	10.6
		L3	8.1	10.2
		L4	10.2	11.6
		L5	13	16.3
Robertson et al. (New Zealand) [29]	100	L1 (L)	10.3	18.6
		L2 (L)	10.3	18.6
		L3 (L)	13	18.6
		L4 (L)	16.3	17.8
		L5 (L)	21.6	18.6
		L1 (R)	9.5	19.9
		L2 (R)	10	18.7
		L3 (R)	12.7	18.7
		L4 (R)	16.5	17.4
		L5 (R)	21.5	19.5
Cook et al. (Australia) [30]	196	L1	7.8	15.1
		L2	8.2	14.6
		L3	10.2	14.4
		L4	12.1	13.6
		L5	16.1	12.9

NS: not specified, L: left pedicle, R: right pedicle, DM: Direct measurement with human cadaveric specimens, RM: radiographic measurements

### Pedicle width

#### L1 pedicle width comparison by geography

The L1 pedicle width was smaller in Americans than in Oceania, larger in Chinese, East and Southeast Asian, West Asian, and South Asian. It was not significantly different from West Asian, South Asian, and Oceania, and statistically different from Chinese, East Asian, and Southeast Asian (Chinese: *p* = 0.011, East and Southeast Asian: *p* = 0.007). The L1 pedicle width was smaller in Chinese than in East and Southeast Asian, West Asian, South Asian, and Oceania, and was not significantly different from East and Southeast Asian, West Asian, and South Asian, and was significantly different compared with Oceania (*p* = 0.001). The width of L1 pedicle in East Asian and Southeast Asian was smaller than that in West Asian, South Asian, and Oceania, and there was no statistical different compared with West Asian and South Asian, but there was a significant difference compared with Oceania (*p* = 0.001). The L1 pedicle width was slightly larger in West Asian than in South Asian and smaller than in Oceania, with no statistical differences. South Asian had a smaller L1 pedicle width than Oceania, and the difference was not significant. Specific data were shown in Additional file 1: Table S1.

#### L2 Pedicle width comparison by geography

The L2 pedicle width in Americans was smaller than in Oceania, larger than in Chinese, East and Southeast Asian, West Asian, and South Asian. It was not significantly different compared with West Asian and South Asian, but significantly different compared with Chinese, East and Southeast Asian, and Oceania (Chinese: *p* = 0.031, East and Southeast Asian:*p* = 0.020, Oceania:*p* = 0.012 for Oceania). The L2 pedicle width of Chinese was smaller than that of East and Southeast Asian, West Asian, South Asian, and Oceania, and there was no significant difference compared with East and Southeast Asian, West Asian, and South Asian, but there was a significant difference compared with Oceania (*p* = 0.001). The width of L2 pedicle in East Asian and Southeast Asian was smaller than that in West Asian, South Asian, and Oceania, and there was no significant difference compared with West Asian and South Asian, but there was a significant difference compared with Oceania (*p* = 0.001). The width of the L2 pedicle in West Asian was smaller than in Oceania and slightly larger than in South Asian, which was not significantly different from South Asian, but significantly different from Oceania (*p* = 0.001). The width of the L2 pedicle was smaller in South Asian than in Oceania, with a significant difference (*p* = 0.001). Specific data were shown in Additional file 2 Table S2.

#### L3 Pedicle width comparison by geography

The width of the L3 pedicle in Americans was smaller than that in Oceania, larger than that in Chinese, East and Southeast Asian, West Asian, and South Asian. It was not significantly different compared with West Asian, but significantly different compared with Chinese, East and Southeast Asian, South Asian, and Oceania (Chinese: *p* = 0.03, East and Southeast Asian: *p* = 0.02, South Asian: *p* = 0.014, Oceania: *p* = 0.01). The width of the L3 pedicle in Chinese was smaller than that in East and Southeast Asian, West Asian, and Oceania, and larger than that in South Asian. There was no significant difference between Chinese and East Asian, West Asian and South Asian, but there was a significant difference between Chinese and Oceania (*p* = 0.001). The width of L3 pedicle in East Asian and Southeast Asian was smaller than that in West Asian and Oceania, larger than that in South Asian, and there was no significant difference compared with West Asian and South Asian, but there was asignificant difference compared with Oceania (*p* = 0.001). The width of the L3 pedicle in West Asian was smaller than that in Oceania and larger than that in South Asian, which was not significantly different from that in South Asian, but significantly different compared with Oceania (*p* = 0.001). The width of the L3 pedicle was significantly smaller in South Asian than in Oceania (*p* = 0.001). Specific data were shown in Additional file 3: Table S3.

#### L4 pedicle width comparison by geography

The width of the L4 pedicle in Americans was smaller than that in Oceania, larger than that in Chinese, East Asian and Southeast Asian, West Asian, and South Asian. It was not significantly different compared with Chinese, West Asian and Oceania, but significantly different compared with East Asian and Southeast Asian and South Asian (East and Southeast Asian: *p* = 0.015, South Asian: *p* = 0.044). The width of the L4 pedicle in Chinese was smaller than that in East and Southeast Asian, West Asian, South Asian, and Oceania, and there was no significant difference. The width of L4 pedicle in East Asia and Southeast Asia was smaller than that in West Asia and Oceania, and larger than that in South Asia. The width of the L4 pedicle in western Asian was smaller than that in Oceania, but larger than that in South Asian, which was not significantly different. The width of the L4 pedicle in South Asian was smaller than that in Oceania, without a significant difference. Specific data were shown in Additional file 4: Table S4.

#### L5 pedicle width comparison by geography

The L5 pedicle width in Americans was smaller than that in Oceania, but larger than that in Chinese, East and Southeast Asian, West Asian, and South Asian. It was not significantly different from Oceania, but significantly different from Chinese, East and Southeast Asian, West Asian, and South Asian (Chinese: *p* = 0.002, East and Southeast Asian: *p* = 0.07, South Asian: *p* = 0.028, West Asian: *p* = 0.07). The L5 pedicle width in Chinese was smaller than that in East and Southeast Asian, West Asian, and Oceania, and larger than that in South Asian, and there was no significant difference between East and Southeast Asian, West Asian, and South Asian, but there was a significant difference compared with Oceania (*p* = 0.001). The L5 pedicle width of East Asian and Southeast Asian was smaller than that of Oceania, larger than that of West Asian and South Asian, and there was no significant difference compared with West Asian and South Asian, but there was a significant difference compared with Oceania (*p* = 0.001). The L5 pedicle width in Western Asian was smaller than that in Oceania, but larger than that in South Asian, with no significant difference compared to South Asian, but a significant difference compared to Oceania (*p* = 0.003). South Asian had a significantly smaller L5 pedicle width than Oceania (*p* = 0.001). Specific data were shown in Additional file 5: Table S5.

#### Mean pedicle width by geography

**Fig. 3:**
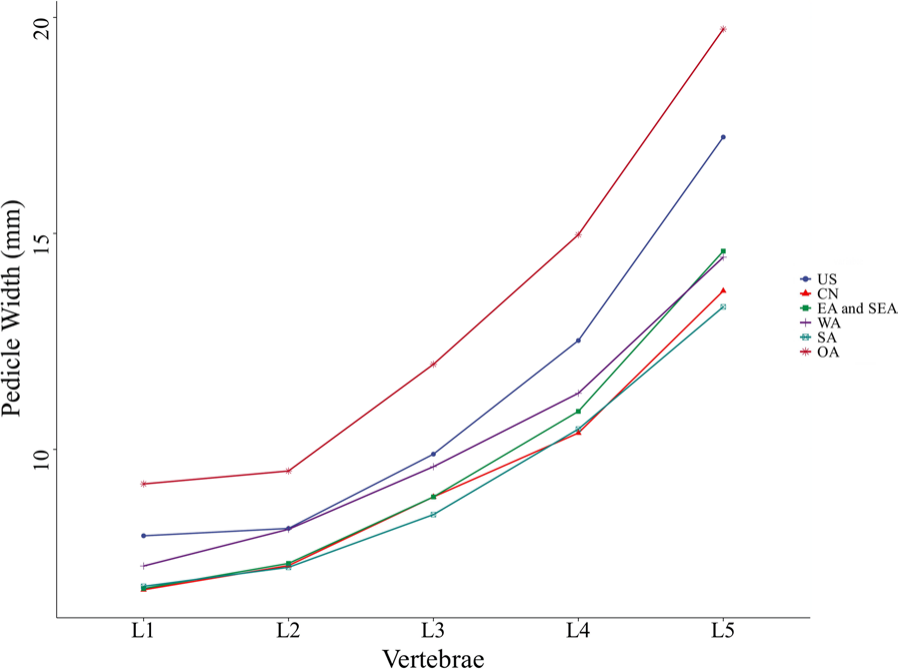
Trends of pedicle width in different regions

According to the data in Table 1, the mean value of pedicle width in different regions was calculated (Table 2). The trend of L1-L5 pedicle width in different regions was plotted according to the data in the table (Fig. 3).

**Table 2:** Mean pedicle width of L1–L5 vertebral body by region (mm)

	US	CN	EA and SEA	WA	SA	OA
L1	8	6.75	6.78	7.3	6.83	9.2
L2	8.17	7.31	7.36	8.15	7.27	9.5
L3	9.89	8.9	8.9	9.6	8.49	11.97
L4	12.52	10.38	10.88	11.3	10.47	14.97
L5	17.23	13.67	14.59	14.45	13.3	19.73

### Pedicle height

#### L1 pedicle height comparison by geography

The L1 pedicle height in Americans was smaller than that in Oceania and larger than that in Chinese, East and Southeast Asian, West Asian, and South Asian, and there was no significant difference compared with Chinese, East and Southeast Asian, West Asian, and Oceania, but there was a statistically significant difference compared with South Asian (*p* = 0.001). The L1 pedicle height in Chinese was higher than that in West Asian, South Asian, East Asian, Southeast Asian, and Oceania. Among them, and there was no significant difference  among the West Asian, East Asian, and Southeast Asian, but there was a significant difference compared with South Asian (*p* = 0.008). The L1 pedicle height in East and Southeast Asian was lower than that in Oceania, higher than that in West Asian and South Asian, and was not significantly different compared with West Asian and Oceania, but significantly different compared with South Asian (*p* = 0.001). Western Asian had a smaller L1 pedicle height than Oceania and larger pedicle height than South Asian, which was not significantly different compared to Oceania, but significantly different compared to South Asian (*p* = 0.002). The L1 pedicle height was significantly lower in South Asian than in Oceania. Specific data were shown in Additional file 6: Table S6.

#### L2 pedicle height comparison by geography

The L2 pedicle height in Americans was smaller than that in Oceania, but larger than that in Chinese, East and Southeast Asian, West Asian, and South Asian, and was not significantly different from that in Chinese, East and Southeast Asian, West Asian, and Oceania, but was significantly different compared to South Asian (*p* = 0.001). The height of the L2 pedicle in Chinese was smaller than that in East and Southeast Asian, West Asian, and Oceania, and higher than that in South Asian. There was no significant difference among Chinese and West Asian, East Asian and Southeast Asian, but there was a statistical different between Chinese and South Asian (*p* = 0.011). The height of L2 pedicle in East and Southeast Asian was smaller than that in West Asian and Oceania, and larger than that in South Asian. Among them  there was not statistically different among East and Southeast Asian and West Asian and Oceania, but there was a significant difference between East and Southeast Asian and South Asian (*p* = 0.001). The L2 pedicle height in West Asian was smaller than in Oceania and larger than in South Asian, which was not significantly different from Oceania, but significantly different from South Asian (*p* = 0.006). The L2 pedicle height was not significantly smaller in South Asian compared to Oceania. Specific data were shown in Additional file 7: Table S7.

#### L3 pedicle height comparison by geography

The L3 pedicle height in Americans was smaller than that in Oceania, but larger than that in Chinese, East and Southeast Asian, West Asian, and South Asian, and was not significantly different from that in Chinese, East and Southeast Asian, West Asian, and Oceania, but was significant compared with South Asian (*p* = 0.001). The height of L3 in Chinese was smaller than that in Oceania, but larger than that in East and Southeast Asia, South Asia, and West Asia, and there was no significant difference compared with East and Southeast Asia, West Asia, and Oceania, but there was a significant difference compared with South Asia (*p* = 0.013). The height of L3 pedicle in East Asian and Southeast Asian was smaller than that in Oceania, but larger than that in South Asian, which was not significantly different compared with Oceania and West Asian, and was statistically different compared with South Asian (*p* = 0.002). The height of the L3 pedicle in West Asian was smaller than that in Oceania and larger than that in South Asian, which was not significantly different compared with Oceania, but significantly different compared with South Asian (*p* = 0.023). The height of the L3 pedicle in South Asian was smaller than that in Oceania, which was not significantly different. Specific data were shown in Additional file 8: Table S8.

#### L4 pedicle height comparison by geography

The L4 pedicle height in Americans was smaller than that in Oceania, and larger than that in Chinese, East and Southeast Asian, West Asian, and South Asian, and was not significantly different from that in Chinese, East and Southeast Asian, West Asian, and Oceania, but was significantly different compared with South Asian (*p* = 0.001). The height of L4 in Chinese was smaller than that in Oceania, but higher than that in East and Southeast Asia, West Asia, and South Asia, and was not significantly different compared with West Asia, East Asia, and Southeast Asia, but was significantly different compared with South Asia. (*p* = 0.027). The height of L4 vertebral pedicle in East Asian and Southeast Asian was smaller than that in West Asian and Oceania, and larger than that in South Asian, with no significant difference. The height of the L4 pedicle in western Asian was smaller than that in Oceania, but larger than that in South Asian, which was not significantly different. South Asian had a smaller L4 pedicle height than the Oceania population, which was not significantly different. Specific data were shown in Additional file 9: Table S9.

#### L5 pedicle height comparison by geography

The L5 pedicle height was smaller in Americans than in Chinese, East and Southeast Asian, South Asian, and Oceania, and larger than in West Asian, which was not significantly different from Chinese, East and Southeast Asian, West Asian, and Oceania, but significantly different compared with South Asian (*p* = 0.013). The height of the L5 pedicle in Chinese was higher than that in East and Southeast Asian, West Asian, South Asian, and Oceania, but it was not significantly different. The height of L5 pedicle in East Asian and Southeast Asian people was smaller than that in South Asian, Oceanic, but larger than that in West Asian people, with no significant difference. The L5 pedicle height was significantly lower in West Asian than in South Asian and Oceania. The L5 pedicle height was not significantly smaller in South Asian compared to Oceania. Specific data were shown in Additional file 10: Table S10.

#### Mean pedicle width by geography

**Fig. 4:**
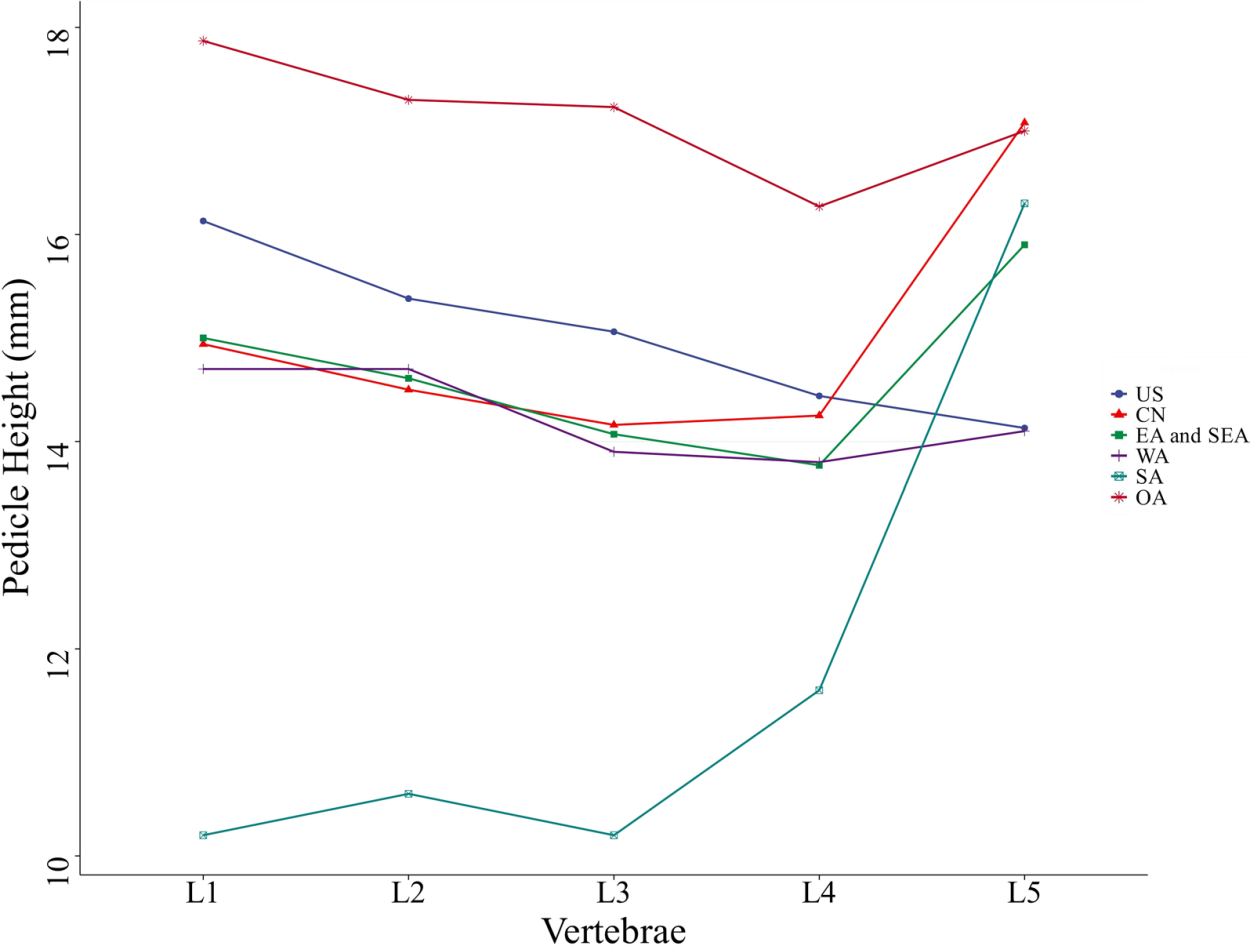
Trends of pedicle height in different regions

According to the data in Table 1, the mean value of pedicle height in different regions was calculated (Table 3). The trend of L1-L5 pedicle height change in different regions was plotted according to the data in the table (Fig. 4).

**Table 3:** Mean pedicle height of L1–L5 vertebral body by region (mm)

	US	CN	EA and SEA	WA	SA	OA
L1	16.13	14.94	15	14.7	10.2	17.87
L2	15.38	14.5	14.61	14.7	10.6	17.3
L3	15.06	14.16	14.07	13.9	10.2	17.23
L4	14.44	14.25	13.77	13.8	11.6	16.27
L5	14.13	17.08	15.9	14.1	16.3	17

## Discussion

The pedicle is the transition site between the vertebral body and the lamina, and Panjiabi describes the shape of the pedicle as a dumbbell-like irregular structure, with the pedicle isthmus as the narrowest part of the pedicle [9] from the coronal plane, the pedicle is an irregular polymorphous tubular structure composed of the outer cortical bone and the inner cancellous bone, and its section is mainly oval, with additional reports of renal, teardrop, and irregular sections [17, 31].

Taking the pedicle width of L1-L5 vertebral body from different regional populations together, we found that Oceania had the largest pedicle width among the countries and regions selected for this study, followed by Americans. West Asian had the largest pedicle width in Asia, followed by East and Southeast Asians. The difference in pedicle width between Chinese and South Asians was not significant, but according to this study, the Chinese had a larger pedicle width. Secondly, the pedicle width of the same regional or national population showed a more consistent and significant increasing trend from L1 to L5, which was consistent with the existing findings [7–30].

Oceania had the largest pedicle height on L1-L4 vertebrae, followed by Americans. In Asia, the pedicle height of West Asians, Chinese, East Asians, and Southeast Asians did not show significant geographical differences, but the pedicle height of South Asians was significantly lower than the first three and was statistically significant. Second, the variation trend of pedicle height at L1-L5 varies among different regions or countries. In this study, we found that there was a significant decreasing trend in pedicle height from the upper lumbar spine to the lower lumbar spine in Americans, but the decreasing trend in pedicle height was not significant in other regions and national populations. Particularly, in this study, there was a significant increase in L5 pedicle height compared with L1-L4 pedicle height in Chinese, South Asian, East Asian, and Southeast Asian patients, but this phenomenon was not obvious in West Asian patients, and Oceania patients and Americans, and the specific reasons were still unclear.

In this study, we found that the width of the pedicle in West Asians was quite different from that in Chinese, East and Southeast Asians, and South Asians and its values were intermediate between Americans, Oceania and Chinese, East and Southeast Asians, and South Asians. We believe this was because West Asia is located in the transition zone between Asia and Europe and has a wider cultural exchange and population movement with both Europe and Asia [32–34]. Albano pointed out that Caucasians have a larger pedicle size compared to Asian, which may be genetically determined [35]. According to the overall regression in the direction of the mean [36], we speculate that the width of the pedicle in West Asians is intermediate between Americans, Oceania and Chinese, East and Southeast Asians, and South Asians, possibly due to the large population movements between West Asia and Europe and Asia. Whereas relatively closed eastern Asia, including China, East and Southeast Asia, South Asia and other continents have relatively few large-scale population movements, more are regional population movements [37–42], so their pedicle widths differ little. Our conjecture also explains in part why there is a significant increase in L5 pedicle height compared with L1-L4 pedicle height in Chinese, East Asian, and Southeast Asian, but this phenomenon is not evident in West Asian, as well as Oceanian, and North American.

Overall speaking, the changing pattern of pedicle height is not exactly similar to that of pedicle width. Although western Asians have a larger pedicle width, their pedicle height is not significantly different from that of Chinese, East Asian, and Southeast Asian, and the pedicle height of South Asians is significantly lower than that of the first three. In response to this phenomenon, further experiments are needed to investigate.

Based on the data of this study, the size and changing rule of pedicle anatomical parameters are not completely similar in different regions or countries of Asia, that is, there are similarities between pedicle anatomical parameters in different regions of Asia, but there are also heterogeneities. It is not rigorous to classify yellow races as one in the entire Asian region as Albano [35], which ignores the objective laws of pedicle anatomical parameter changes in different Asian regions.

There are still some shortcomings in this article. The purpose of this article is to statistically analyze the pedicle height and width in people from different regions through published articles, and to derive the average pedicle height and width in people from different regions, and to make a cross-sectional comparison of the vertebral pedicle width and height in different regions on this basis. In order to reduce the risk of data bias, some of the older literature reporting pedicle width and height was selected for this article. Most of the literature selected for this study did not report the gender, race, or underlying skeletal disorders of the subjects measured, and some of the literature reported only the mean age of the subjects measured rather than the age range. Therefore, this paper does not group the subjects by race, gender, or age, but rather derives the mean pedicle height and width in a given region regardless of age, gender or race based on the existing literature.

Based on the pedicle width and height data in published articles, mean pedicle height and width in different regions or countries were obtained, but different genders, ages, heights, and weights may have different effects on the pedicle. In the actual clinical individualized treatment, the data and results of this study cannot be completely relied on. Surgeons should carry out individualized CT or MR scanning of patients before surgery to comprehensively master the morphological parameters of pedicles in patients, lay an objective foundation for the selection of screw diameter, and reduce the possibility of vascular destruction caused by pedicle breakage.

## Conclusions

Based on the data of pedicle width and height in previous studies, the pedicle width and height in different regions or countries were compared transversely, and the similarities, differences and changes of pedicle height and width in different regions were obtained. Overall speaking, there is a relationship between the morphological characteristics of the human lumbar pedicle and geographical location. People in geographically close areas showed similar lumbar pedicle morphology and changing pattern even they belonged to different ethnic groups, and it is tentatively assumed that this may be related to inter-regional population movements.

## Supplementary Information

**Additional file 1.** Comparison of L1 pedicle width in different regions (LSD test).

**Additional file 2.** Comparison of L2 pedicle width in different regions (LSD test).

**Additional file 3.** Comparison of L3 pedicle width in different regions (LSD test).

**Additional file 4.** Comparison of L4 pedicle width in different regions (T2 test).

**Additional file 5.** Comparison of L5 pedicle width in different regions (LSD test).

**Additional file 6.** Comparison of L1 pedicle height in different regions (T2 test).

**Additional file 7.** Comparison of L2 pedicle height in different regions (T2 test).

**Additional file 8.** Comparison of L3 pedicle height in different regions (T2 test).

**Additional file 9.** Comparison of L4 pedicle height in different regions (T2 test).

**Additional file 10.** Comparison of L5 pedicle height in different regions (T2 test).

## Data Availability

All datasets generated for this study are included in the article/Supplementary Material.
